# Red Card for Pathogens: Phytoalexins in Sorghum and Maize

**DOI:** 10.3390/molecules19079114

**Published:** 2014-06-30

**Authors:** Alana Poloni, Jan Schirawski

**Affiliations:** Department of Microbial Genetics, Institute of Applied Microbiology, Aachen Biology and Biotechnology, RWTH Aachen University, Worringerweg 1, Aachen 52074, Germany; E-Mail: alana.poloni@rwth-aachen.de

**Keywords:** luteolinidin, apigeninidin, kauralexin, zealexin, DIMBOA, HDMBOA, biosynthesis, regulation, sorghum, maize

## Abstract

Cereal crop plants such as maize and sorghum are constantly being attacked by a great variety of pathogens that cause large economic losses. Plants protect themselves against pathogens by synthesizing antimicrobial compounds, which include phytoalexins. In this review we summarize the current knowledge on phytoalexins produced by sorghum (luteolinidin, apigeninidin) and maize (zealexin, kauralexin, DIMBOA and HDMBOA). For these molecules, we highlight biosynthetic pathways, known intermediates, proposed enzymes, and mechanisms of elicitation. Finally, we discuss the involvement of phytoalexins in plant resistance and their possible application in technology, medicine and agriculture. For those whose world is round we tried to set the scene in the context of a hypothetical football game in which pathogens fight with phytoalexins on the different playing fields provided by maize and sorghum.

## 1. What’s at Stake: Maize and Sorghum

The world population increases by around 1% every year [1]. The rising number of people necessitates an ongoing expansion in food production. Currently, the largest part of food supply stems from the production of cereal crops such as maize (*Zea mays*) and sorghum (*Sorghum biolor*) [2]. To be sufficient to feed the population, studies indicate that global crop production needs to double by 2050. However, the production of maize, the most cultivated cereal in the world, is only increasing at a rate of 1.6% per year, while the rate increase necessary to match world population growth would be 2.4% [3].

Maize is the most cultivated cereal in the world, with 875 million tons produced in 2012 [2] and a worldwide consumption of more than 116 million tons. Maize ranks highest in net energy content and lowest in protein and fiber content relative to other cereals [2]. The plant is utilized mainly for human and animal livestock feed, but is also used for non-food products and for generation of bioenergy, for example in agricultural biogas production [4].

Sorghum is the fifth most highly produced crop and total production reached 58 million tons in 2012 [2]. The predominantly cultivated sorghum plant (*Sorghum bicolor*) exists in several subspecies or races with different morphological and physiological characteristics [5]. Among the main advantages of this plant are its high draught and heat tolerance, its high sugar content and the high yields of forage biomass that can be obtained per unit of land. Due to these advantages, sorghum is cultivated especially in hot and arid regions, like Nigeria and India, as well as USA and China [2]. Although sorghum is also used for industrial purposes, such as the generation of fiber, paper and ethanol, its main use is still for feed and food. Recently, the plant became even more important for the food industry, because it can be used to produce gluten-free products [6].

## 2. Setting the Game: Pathogens Attack

Despite the importance of sorghum and maize for agriculture and industry, a part of the harvest is lost either during cultivation or storage. Losses during cultivation are mainly due to the action of different pathogens, like bacteria, fungi, oomycetes, nematodes, parasitic plants and viruses. Pathogen attack affect the amount and quality of the grains, making them unsuitable for consumption. Microbial pathogens are difficult to control since they have genetically diverse populations that quickly adapt to changing environments and can readily break resistance of potential host plants [7]. However, although many pathogens exist, generally only a few species or a few strains of a given species are able to successfully infect a certain plant host. For example, the smut fungus *Ustilago maydis* causes tumors in leaves and inflorescences of maize, while the close relatives *Ustilago hordei* and *Sporisorium scitamineum* attack barley and sugarcane, respectively [8,9]. A more extreme example of host specificity occurs in the smut fungus *Sporisorium reilianum*, where two *formae speciales* exhibit different host preferences: *S. reilianum f. sp. reilianum* is an efficient sorghum pathogen, while *S. reilianum f. sp. zeae* is a maize pathogen that is unable to cause disease on sorghum [10].

In order to deal with or avoid the damaging effects of the pathogens, plants possess intricate defense mechanisms. Defense responses include the generation of reactive oxygen species and callose deposition at the point of entry, lignification of colonized plant tissues, activation of defense genes and production of antimicrobial substances [11,12,13]. Among the plant-produced antimicrobials that are induced upon pathogen attack are the phytoalexins, which are compounds of low molecular weight that are induced by stress [14,15,16].

In spite of our increased understanding of plant health and disease, millions of dollars’ worth of harvest are still lost every year due to plant pathogens. In the search for effective plant protection measures, there is a renewed interest in the study of phytoalexins, since they are natural compounds with the potential to effectively protect our crops against pathogen attack. In this review, we focus on phytoalexins known to be produced by sorghum and maize. We summarize what is known about their biosynthetic pathways and their mechanisms of elicitation. Finally, we discuss the use of phytoalexins in agriculture, human health and industry.

## 3. The Fullback: Phytoalexins in Sorghum

**Figure 1:**
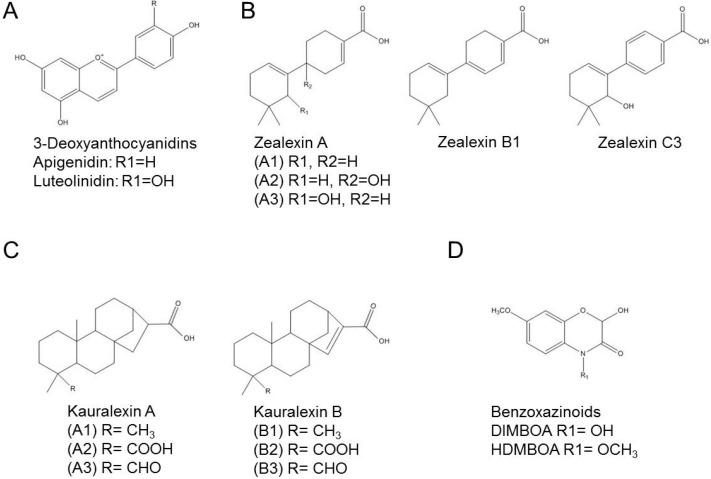
Structural formula of phytoalexins produced by sorghum and maize. (**A**) 3-Deoxyanthocyanidins produced by sorghum; (**B**–**D**) Phytoalexins produced by maize; (**B**) Zealexins; (**C**) Kauralexins; (**D**) Benzoxazinoids. Structures adapted from [23,24].

Sorghum produces two distinct 3-deoxyanthocyanidin phytoalexins, known as apigeninidin (2-(4-hydroxyphenyl)benzopyrilium chloride) and luteolinidin (2-(3,4-dihydroxyphenyl)chromenylium-5,7-diol) (Figure 1A), in addition to a variety of derivatives, like 5-methoxy-luteolinidin, caffeic acid ester of arabinosyl-5-O-apigeninidin, and 7-methoxyapigeninidin [17,18]. A mixture of these characteristic reddish- and orange-colored compounds are known to be synthesized in the cytoplasm of epidermal sorghum cells infected with *Colletotrichum sublineolum* where they accumulate in initially colorless inclusion bodies. These inclusion bodies migrate to the infection zone, where they first accumulate and become pigmented, then lose their spherical shape and release their red contents at the infection site [19,20]. Accumulation of the 3-deoxyanthocyanidinsoccurs much faster in pathogen-challenged cells of resistant cultivars than of susceptible ones [21,22], suggesting that early phytoalexin accumulation is important to prevent proliferation and spread of fungal hyphae.

The measurement of the phytoalexin precursors naringenin and eriodictyol shows that they do not accumulate to measurable constitutive levels in unchallenged *S. bicolor* [25]. The low concentration or the rapid turnover of precursor compounds suggests *de novo* synthesis upon challenge of the plant by a fungal intruder [25]. In line with this hypothesis, genes of the 3-deoxyanthocyanidin biosynthesis phenylalanine ammonia lyase, chalcone synthase, and dihydroflavonol 4-reductase were induced when sorghum was challenged with the maize pathogens *Bipolaris maydis* or *S. reilianum f. sp. zeae* but not when challenged with the sorghum pathogen *S. reilianum f. sp. reilianum* [10,26]. 

## 4. Additional Defense Players: Versatile Plant Defense Responses

There is evidence that 3-deoxyanthocyanidin induction is not the only level of defense used by sorghum upon pathogen attack. In sorghum seedlings inoculated with *Fusarium proliferatum* and *Fusarium thapsinum*, the increase in apigeninidin and luteolinidin levels was accompanied by increased concentrations of peroxidases, beta-1,3-glucanases and chitinases [27]. In sorghum seedlings inoculated with *Cochliobolus heterostrophus*, in addition to phytoalexin accumulation, a fast and coordinated accumulation of *PR-10* and chalcone synthase transcripts was observed. This accumulation was delayed in sorghum seedlings inoculated with the sorghum pathogen *C. sublineolum* [28,29]. Recently, the whole transcriptome of sorghum inoculated with the necrotroph *Bipolaris sorghicola* was analyzed. In addition to the up-regulation of genes encoding key enzymes for phytoalexin biosynthesis, many other plant genes with a suspected role in defense were up-regulated, which included genes encoding plant receptors, genes involved in MAPK cascades and Calcium signaling, transcription factors and genes involved in downstream responses (peroxidases, PR proteins and genes implicated in biosynthesis of lignin) [30,31].

## 5. Preparation Phase: Phytoalexin Biosynthesis

**Figure 2:**
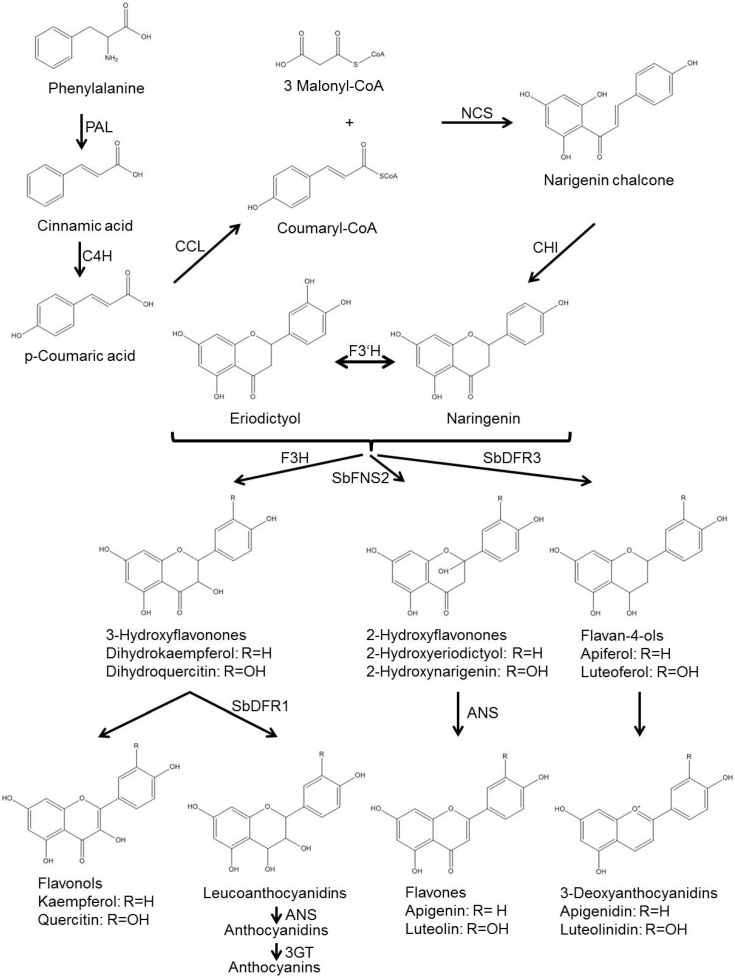
Biosynthetic pathway of 3-deoxyanthocyanidins in sorghum. Structures of intermediates and products are shown. Where known, enzyme classes are indicated. In many cases the specific enzyme has not been identified yet. ANS, anthocyanidin synthase; C4H, cinnamate-4-hydroxylase; CCL, coumaryl-CoA ligase; CHI, chalcone isomerase; F3'H, flavanone-3'-hydroxylase; F3H, flavanone-3-hydroxylase; NCS, Naringenin chalcone synthase; PAL, phenylalanine ammonia lyase; SbDFR1, dihydroflavonol 4-reductase 1; SbDFR3, dihydroflavonol 4-reductase 3; SbFNS2, flavone synthase 2. Pathway adapted from [33,35].

Biosynthesis of the 3-deoxyanthocyanidin phytoalexins is independent of light and occurs in the dark, in contrast to the biosynthesis of anthocyanins that is light dependent [32]. Biosynthesis of the 3-deoxyanthocyanidins luteolinidin and apigeninidin, of the flavones luteolin and apigenin, and the leucoanthocyanidins and anthocyanins occurs via common and specific pathway steps [17,33,34]. Commons steps include the formation of *p*-coumaryl CoA, that is generated from phenylalanine via the action of the enzymes phenylalanine ammonia lyase (PAL) to synthesize cinnamic acid, cinnamate-4-hydroxylase (C4H) to synthesize *p*-coumaric acid, and coumaryl CoA ligase (CCL) for generation of *p*-coumaryl CoA. *p*-Coumaryl CoA is the substrate of the enzyme chalcone synthase (CHS) [33], which catalyzes the condensation of *p*-coumaryl CoA and three molecules of malonyl CoA form naringenin chalcone that is converted to naringenin by a chalcone isomerase (CHI) (Figure 2).

It is from the flavanone naringenin that the biosynthesis pathways of anthocyanin, flavone and 3-deoxyanthocyanidin split [33]. The flavones apigenin and luteolin are generated from naringenin and the related flavanon eriodictyol that is likely generated from naringenin via a flavonoid-3'-hydroxylase (F3'H). It is the enzyme flavon synthase (FNS) that catalyzes both hydroxylation at C-2 and abstraction of water [33]. In the anthocyanin pathway, naringenin and related flavanones are hydroxylated at C-3 by flavanone-3-hydroxylase (F3H), followed by an NADPH-dependent reduction of the C-4 carbonyl group by dihydroflavonol 4-reductase (SbDFR1), and the action of the anthocyanidin synthase (ANS), that abstracts water leaving a double bond between C-3 and C-4. The unstable anthocyanidins are then converted to the stable anthocyanins by a flavonol 3-*O*-glucosyltransferase (3GT) that attaches a glucose molecule to the C-3 hydroxyl group [33]. In contrast, for biosynthesis of the 3-deoxyanthocyanidins, naringenin and eriodictyol are direct targets of NADPH-dependent reduction of the C-4 carbonyl group by a dihydroflavonol 4-reductase (SbDFR3), that has been shown to be a different enzyme than the dihydroflavonol 4-reductase SbDFR1 involved in anthocyanin biosynthesis [33]. The generated luteoferol and apiferol (that might also be generated from luteoferol via an F3'H) are likely reduced by an unidentified anthocyanidin synthase that removes the C-4 hydroxyl group leaving a double bond between C-3 and C-4 and creating the 3-deoxyanthocyanidins apigeninidin and luteolinidin [33].

A candidate gene, *Sb06g029550*, has been identified as induced upon *B. sorghicola* infection of sorghum [30], which might correspond to the unidentified anthocyanidin synthase responsible for generation of apigeninidin and luteolinidin from apiferol and luteoferol. Gene expression of this enzyme coincided with the accumulation of apigeninidin detected during *B. sorghicola* infection [30]. Fungal inoculation with the non-sorghum pathogen *Cochliobolus heterostrophus* shifts metabolic flux away from anthocyanin synthesis towards 3-deoxyanthocyanidin synthesis [36]. This is achieved by induction of PAL and CHS presumably leading to an increased synthesis of naringenin, as well as SbDFR3 and the ANS involved in this pathway [33,36], and simultaneous repression of the anthocyanin biosynthesis genes *F3H*, *SbDFR1* and *ANS*. Both SbDFR1 and SbDFR3 are able to convert flavanones to flavan-4-ols *in vitro* [33]. However, only *SbDFR1* is up-regulated under light-induced anthocyanin biosynthesis, while only *SbDFR3* is up-regulated during 3-deoxyanthocyanidin biosynthesis [33]. Transcriptome data of sorghum infected with *B. sorghicola* identified four putative paralogs of *DFR* genes, but only one was up-regulated and had a similar sequence as *SbDFR3*, suggesting that this was the one involved in the reduction of the C-4 group of narigenin [30].

## 6. Training with New Methods: Unknown Biosynthesis Genes

Although the enzyme class necessary for the specific biosynthetic steps can be easily predicted, the redundancy of the enzyme complement makes it difficult to predict which of the *S. bicolor* genes encode the relevant one for a specific catalytic step. For example, first analysis of the genome sequence of *S. bicolor* identified eight genes potentially encoding chalcone synthases [37]. Seven of these (SbCHS1 to SbCHS7) are highly conserved, while one (SbCHS8) shows an amino acid identity of only about 82% to the other seven enzymes [38]. Of the eight *CHS* genes, *SbCHS8* was overrepresented in a cDNA library prepared from *C. sublineolum*-inoculated sorghum, suggesting that SbCHS8 was involved in 3-deoxyanthocyanidin biosynthesis [38]. However, *SbCHS8* was found to encode a functional stilbene synthase (STS) that is activated during both host and non-host responses, being therefore renamed to *SbSTS1* [39]. In contrast, *SbCHS2* could be shown to encode a typical chalcone synthase that is able to synthesize naringenin chalcone *in vitro* [39]. It is possible that more than one of the remaining seven chalcone synthases of sorghum is responsible for production of naringenin chalcone during pathogen attack and that there are even more chalcone synthases present. Transcriptome sequencing of sorghum infected with *B. sorghicola* revealed nine *SbCHS* genes, of which six were up-regulated [30].

The genome of *S. bicolor* shows presence of three unique sorghum flavonoid 3'-hydroxylases [37]. Of these, *SbF3'H1* expression was induced during light-induced anthocyanin accumulation, while *SbF3'H2* expression was induced during pathogen-specific 3-deoxyanthocyanidin synthesis. Expression of *SbF3'H3* was not detected under these conditions, leaving its potential *in vivo* function unexplained [23]. Identification of the specific flavone synthase involved in flavone biosynthesis was more straightforward. Investigation of pathogen-inducible gene expression identified a cytochrome P450 protein that was shown to generate flavones from flavanones via formation of 2-hydroxyflavanones. The gene is located in single-copy on chromosome 2 and was named *SbFNS2* [35].

## 7. Kick-Off: Phytoalexin Induction

The 3-deoxyanthocyanidins are synthesized in sorghum in response to different stimuli [40]. The study of phytoalexin induction by fungal intruders was pioneered by Nicholson and coworkers [19]. The authors inoculated sorghum leaves with the non-sorghum pathogen *Helminthosporium maydis* and the sorghum pathogen *Colletotrichum graminicola*. They tested two sorghum cultivars, BR54 (resistant) and P721N (susceptible) and identified the phytoalexins apigenidin and luteolinidin, of which apigenidin accumulated in both cultivars, while luteolinidin was present only in the resistant one. Both compounds were active in inhibiting germling development in *H. maydis* and elongation of *C. graminicola* germ-tubes *in vitro* [19]. Apigeninidin was also shown to inhibit growth of the gram-positive bacteria *Bacillus cereus*, *Staphylococcus aureus*, *Staphylococcus epidermidis*, and *Streptococcus faecalis*, as well as the Gram-negative bacteria *Escherichia coli*, *Serratia marcescens*, and *Shigella flexneri* [41].

The order and timing of appearance of the different phytoalexins in sorghum seems to be carefully choreographed. Sorghum leaves inoculated with the maize pathogen *B. maydis* show detectable levels of apigeninidin first at 10 h post inoculation (hpi), of luteolinidin and apigeninidin-5-*O*-arabinoside at 14 hpi, of luteolinidin-5-methylether at 18 hpi and apigeninidin-7-methylether at 20 hpi. At 24 hpi, the levels of apigeninidin and luteolinidin were similar, but by 48 hpi the amounts of luteolinidin reached the double of apigenidin [17].

**Figure 3:**
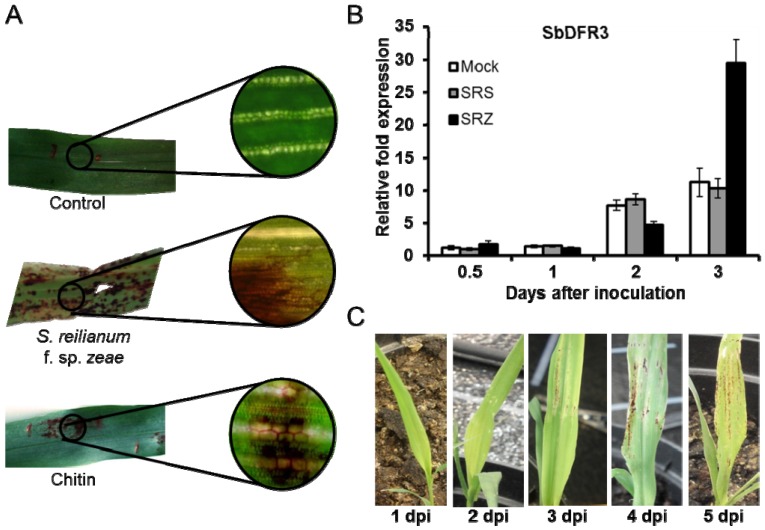
Deposition of 3-deoxyanthocyanidins in sorghum during interaction with the smut fungus *Sporisorium reilianum*. (**A**) Sorghum leaves infiltrated with water (control), infected with *S. reilianum f. sp. zeae* or infiltrated with chitin. The latter treatments lead to appearance of spots with a characteristic red color indicating phytoalexin production; (**B**) Quantitative RT-PCR of sorghum samples inoculated with water (Mock), *S. reilianum f. sp. reilainum* (SRS) or *S. reilianum f. sp. zeae* (SRZ). Sorghum leaves were collected at 0.5, 1, 2 and 3 days post infection (dpi). Up-regulation of the gene *SbDFR3* was observed only for samples infected with SRZ; (**C**) Sorghum leaves infected with SRZ showing the emergence of red color at 3 dpi, which gets more intense with time.

Differential induction of phytoalexins may support host specificity of the two *formae speciales* of *S. reilianum*. Inoculation of sorghum with the maize-pathogenic S*. reilianum f. sp. zeae* resulted in a strong deposition of luteolinidin and apigeninidin (Figure 3A). In contrast, no phytoalexins were generated when sorghum was inoculated with the sorghum-pathogenic *S. reilianum f. sp. reilianum* [10]. Quantitative RT-PCR confirmed increased expression of the phytoalexin biosynthesis gene *SbDFR3* in samples infected with *S. reilianum f. sp. zeae*, while in samples infected with *S. reilianum f. sp. reilianum* the levels were similar to control samples (Figure 3B). Phytoalexins were visible on leaves at 3 dpi as dark red-colored stains which increased in number and size at later time points (Figure 3C). *In vitro*-assays demonstrated that luteolinidin but not apigeninidin was able to slow growth of *S. reilianum* [10]. The concentration of phytoalexins in infected host cells was estimated to be between 0.48 and 1.20 ng of luteolindin and 0.24 to 0.91 ng of apigeninidin per cell [42], which is more than what is necessary for *in vitro* toxicity. Interestingly, *in vitro* growth of both *S. reilianum f. sp. zeae* and *S. reilianum f. sp. reilianum* was equally affected by luteolinidin [10], suggesting that the active phytoalexin is not induced when sorghum is colonized by *S. reilianum f. sp. reilianum*.

## 8. Changing the Pitch: Phytoalexins in Maize

In maize, phytoalexins are represented by terpenoids, which include zealexins and kauralexins and benzoxazinoids, represented by DIMBOA and HDMBOA. Zealexins were recently identified as a group of acidic sesquiterpenoids that are related to β-macrocarpene (4',5,5-trimethyl-1,1'-bis(cyclo- hexane)-1,3'-diene) [43]. The group contains at least five different compounds, zealexin A1, A2, A3, B1 and C3 (Figure 1B), that accumulate to very high levels of about 800 µg/g in *Fusarium graminearum*-infected maize [43]. Nine additional related compounds have been detected by expanded GC/(+)CI-MS but their exact identity is not yet known [43]. High concentrations of zealexins were also found in maize challenged with other fungal pathogens, such as *Aspergillus flavus* and *Rhizopus microsporus* [43]. In vitro toxicity tests showed that zealexin A1 and A3 (but not A2) inhibited growth of *A. flavus* and *F. graminearum*, and that A1 was also effective against *R. microsporus* [43].

Zealexin biosynthesis likely involves terpene synthases 6 and 11 (TPS6 and TPS11). These very similar enzymes were shown to convert farnesyl pyrophosphate to (*S*)-β-macrocarpene via an (*S*)-β-bisabolene intermediate [44,45]. Accordingly, *TPS6* and *TPS11* are transcriptionally induced prior to the rise in zealexin concentration and figure among the most highly induced maize genes upon *F. graminearum* infection [43]. *TPS6* and *TPS11* were also found to be highly induced in maize leaves colonized by the maize smut fungus *U. maydis* [46,47] and in maize inflorescences colonized by the head smut fungus *S. reilianum f. sp. zeae* [48]. Co-silencing of *TPS6* and *TPS11* led to increased susceptibility to *U. maydis* [49]. This indicates that the zealexins are terpenoid phytoalexins produced by maize as part of its defense against fungal intruders.

In addition to zealexin, maize can produce kauralexins that are *ent*-kaurane related diterpenoid phytoalexins. So far, six kauralexins are known: kauralexin A1 (*ent*-kauran-17-oic acid), A2 (*ent*-kauran-17,19-dioic acid), A3 (*ent*-kaur-19-al-17-oic acid), B1 (*ent*-kaur-15-en-17-oic acid), B2 (*ent*-kaur-15-en-17,19-dioic acid), and B3 (*ent*-kaur-15-en-19-al-17-oic acid) (Figure 1C) [50]. Before the accumulation of kauralexins, a strong up-regulation of the *AN2* gene that encodes the enzyme *ent*-copalyl diphosphate synthase anther ear 2 was observed in maize infected with *F. graminearum* [51]. This gene was identified as an ortholog of rice genes encoding ent-copalyl diphosphate synthases that supply precursors to diterpenoid phytoalexins [50,51]. Kauralexins also accumulated in maize stems colonized by the pathogens *R. microsporus* and *C. graminicola* [50]. From the six detected *ent-*kaurane–related diterpenoids, kauralexin A3 and B3 presented antimicrobial activity against *R. microsporus* and *C. graminicola* when used in relevant concentrations [50].

Kauralexin biosynthesis seems to be regulated by phytohormones. Application of a combination of jasmonic acid (JA) and ethylene to maize plants was sufficient to induce kauralexin generation [50]. The gene *AN2* was highly expressed at 24 h after attack by the insect *Ostrinia nubilalis*, which was accompanied by an increase in the concentrations of JA and ethylene together with the expression of allene oxide synthase and 1-aminocyclopropane-1-carboxylic acid oxidase genes, key enzymes involved in biosynthesis of these hormones [52]. At 48 h post attack, plants accumulated kauralexins at higher concentration than at 24 h [53].

Microarray analysis in *Fusarium graminearum*-infected maize showed that not only *AN2* was up-regulated, but also *TPS6* and *TPS11* that are involved in zealexin biosynthesis [43]. In maize roots infected with the oomycete *Phytophthora cinnamori,* *TPS11* and *AN2* were two of the most highly up-regulated genes [54]. These results demonstrate that zealexins and kauralexins are co-inducted and co-produced in maize [43].

In addition to zealexins and kauralexins, benzoxazinoid hydroxamic acids can be produced in maize leaves [55], namely HDMBOA-Glc (2-hydroxy-4,7-dimethoxy-1,4-benzoxazin-3-one-glucoside) and DIMBOA-Glc (4-dihydroxy-7-methoxy-1,4-benzoxazin-3-one-glucoside, Figure 1D), in addition to precursors and variants that include HMBOA-Glc (2-hydroxy-7-methoxy-1,4-benzoxazin-3-one glucoside), DIM2BOA-Glc (2,4-dihydroxy-7,8-dimethoxy-1,4-benzoxazin-3-one glucoside), HBOA (2-hydroxy-2*H*-1,4-benzoxazin-3(4*H*)-one), DIBOA (2,4-dihydroxy-2*H*-1,4-benzoxazin-3(4*H*)-one) and TRIBOA (2,4,7-trihydroxy-2*H*-1,4-benzoxazin-3(4H)-one). DIMBOA-Glc is predominant in maize seedlings, and its levels decrease as the plants get older [56]. Therefore, these compounds have been described as phytoanticipins. However, additional DIMBOA can be synthesized upon pathogen infection [57], and this pool of DIMBOA has to be considered as phytoalexins. The benzoxazinoid biosynthetic pathway has been elucidated and involves the generation of indole by an indole-3-glycerol phosphate lyase (BENZOXAZINE-DEFICIENT1 [BX1]), and subsequent production of indolin-2-one by BX2, 3-hydroxyindolin-2-one by BX3, HBOA by BX4, DIBOA by BX5, DIBOA-Glc by BX8 and BX9, TRIBOA-Glc by BX6 and finally DIMBOA-Glc by BX7 [58,59].

Insect feeding triggers the conversion of DIMBOA-Glc to HDMBOA-Glc and increases plant resistance against some pathogens, but can also be associated with susceptibility in selected examples [52,53,55]. Maize inoculation with the mycorrhizal fungus *Glomus mosseae* increased the production of DIMBOA and reduced disease caused by *Rhizoctonia solani*. The defense genes *PR2a*, *PAL*, and *AOS* were up regulated, together with *BX9*, one of the key genes in DIMBOA biosynthesis [60]. During herbivory with *O. nubilalis*, a decrease of DIMBOA-Glc was observed, while both HDMBOA-Glc and plant resistance increased [52]. The insect *Spodoptera littoralis* triggered accumulation of DIMBOA that was attributed to *de novo* synthesis [61]. HDMBOA-Glc also accumulated in response to treatment with JA, pathogen infection, and herbivory [62]. The smut fungus *U. maydis* induced DIMBOA in maize, but the fungus was resistant to this compound [46]. Another experiment using *U. maydis* detected differences in gene expression for *BX1*, *BX2*, *BX5* and *BX8*, but none for *BX3*, *BX4*, *BX6* and *BX7* [58]. HDMBOA-Glc accumulated in tissues infected with *F. graminearum* in wild-type and benzoxazine-deficient1 (bx1) mutant lines, suggesting that the Bx1 gene and the presence of DIMBOA-Glc were not necessary for HDMBOA-Glc biosynthesis [43]. A gene similar to *BX1*, called *IGL*, also encodes an indole-3-glycerol phosphate lyase, and could be a potential candidate for the synthesis of HDMBOA-Glc in *bx1* plants [59].

## 9. Know the Rules of the Game: Elicitation and Regulation of Phytoalexin Biosynthesis

Phytoalexins in maize and sorghum are induced during pathogen infection, which suggests that molecules originating from the pathogen or generated during host-pathogen interaction act as elicitors [15]. Although non-pathogens and even several pathogens induce phytoalexins in maize and sorghum, only very little is known about the eliciting molecules. Specific elicitors may include avirulence proteins or effectors that are produced by the pathogen during infection, while pathogen associated molecular patterns (PAMPs) such as conserved proteins, glycoproteins, oligosaccharides and fatty acids could serve as more general elicitors. PAMPs, including flagellin and lipopolysaccharide of bacteria, and chitin, chitosan and β-glucan of fungi, are documented as elicitors of phytoalexins in several plant species, like tobacco, rice, soybean, lemon and *Arabidopsis* [63,64]. In maize, treatment of wounded stems with the PAMP polygalacturonase from *Rhizopus sp*. resulted in kauralexin accumulation within 24 h [50]. Fungal β-1,3-glucan also serves as PAMP, as shown by over-expression of the glucan synthase CgGLS1 of *C. graminicola* in maize, which led to up-regulation of the terpene synthase genes potentially involved in phytoalexin biosynthesis as well as to a reduction in pathogen spread [65]. Interestingly, *C. graminicola* decreases its β-1,3-glucan production during the first hours after plant penetration, which presumably leads to avoidance of PAMP-elicited plant defense responses during its biotrophic growth phase [65].

In sorghum, infiltration of leaves with a chitin solution led to a strong induction of phytoalexins, revealing the potential of this PAMP as an elicitor (Figure 3A; Poloni and J. Schirawski, unpublished). Different preparations of *Saccharomyces cerevisiae* generated by heat inactivation and extracted with ethanol were also able to induce phytoalexins when incubated with sorghum seedlings, and the response increased with higher concentrations of proteins in the elicitor sample [66]. Carbohydrates and peptides extracted from conidia of *C. graminicola* also induced phytoalexin deposition in sorghum mesocotyls [67].

While in *Arabidopsis* and rice several signaling components involved in phytoalexin biosynthesis have been identified (for example, camalexin production in *Arabidopsis* is regulated by mitogen-activated protein kinases (MAPK) AtMPK3, AtMPK6 and AtMPK4, and diterpenoid phytoalexins in rice are regulated via the MAPKs OsMPK3 and OsMPK6 [68], very little is known about regulation of phytoalexin biosynthesis in maize and sorghum. In sorghum, the *Y1* (*YELLOW SEED1*) gene that encodes a MYB transcription factor involved in regulation of phlobaphene biosynthesis [69] was also shown to regulate biosynthesis of 3-deoxyanthocyanidins [70]. *Y1* null alleles do not accumulate 3-deoxyanthocyanidins when challenged with the non-pathogenic fungus *C. heterostrophus*, and also show greater susceptibility to the pathogenic fungus *C. sublineolum*, demonstrating that the accumulation of 3-deoxyanthocyanidins and resistance to *C. sublineolum* in sorghum require a functional *Y1* gene [70].

In maize, a number of genes have been identified to be involved in regulation of anthocyanin biosynthesis. Plants with a mutated *PAC1* gene (*PALE ALEURONE COLOR1*) exhibited reduced levels of anthocyanins and reduced transcript levels of the anthocyanin biosynthetic genes, while transcript levels of the regulatory genes *B* and *C1* did not decrease [71]. The product of the *R* gene induces phytoalexins through regulation of a CHS encoded by *C2*, a DFR encoded by *A1*, and 3GT encoded by *Bz1*. For activation of anthocyanin biosynthesis, one protein of the bHLH-transcription factors B and R, and one protein of the Myb-transcription factors C1 and P1 needs to be expressed [72]. Maize cells overexpressing C1 and R accumulated anthocyanins, while cells overexpressing P accumulated 3-deoxyflavonoids [73]. In sorghum, anthocyanin biosynthesis has not been elucidated. Because of partial overlap in precursors, generation of anthocyanins and 3-deoxyanthocyanidin phytoalexins in sorghum may be regulated by the same proteins. As homolog of the maize *P1* gene, the sorghum *Y1* gene (see above) was identified. The encoded MYB-type regulatory protein controls expression of a F3′H [74]. However, in sorghum leaves infected with *B. sorghicola*, *Y1* was completely suppressed in both infected and control samples, while the genes encoding *CHS*, *CHI*, and *F3′H* were differentially expressed [30]. This suggests that additional regulators are active in sorghum.

Phytoalexin biosynthesis in sorghum is regulated via hormones. In sorghum roots, JA stimulates phytoalexin deposition while salyclic acid (SA) had an inhibitory function [33]. In contrast, microarray analysis of sorghum gene expression in response to SA and methyl jasmonate (MeJA) identified many phytoalexin biosynthesis genes as induced by both SA and MeJA. These included genes encoding PAL, C4H, cinnamyl alcohol dehydrogenase, cinnamoyl-CoA reductase, CHS, chalcone-flavanone isomerase, flavanone 3-hydroxylase, dihydroflavonal-4-reductase, isoflavone reductase, and leucoanthocyanidin dioxygenase [75].

## 10. Scoring Goals on other Fields: Applications of Phytoalexins

In addition to up-regulation of phytoalexin biosynthesis genes together with other plant defense genes, a positive relationship between the presence of phytoalexins and non-virulence of certain pathogens was established and was supported by *in vitro* inhibition tests [10,20,35,76,77]. However, in some plant pathogen interactions, fungal strategies for overcoming the deleterious effects of phytoalexins were discovered and the responsible genes identified. For example, the fungal pathogens *Leptosphaeria maculans* and *Alternaria brassicicola* detoxify the phytoalexin brassinin present in crucifers using brassinin oxidases that hydrolyze the dithiocarbamate group of brassinin to generate the non-toxic (1H-indol-3-yl)methanamine [78,79]. Similarly, the pea pathogen *Nectria haematococca* detoxifies the phytoalexin pisatin using pisatin demethylase [80,81]. In potato, *Gibberella pulicaris* is successful during infection due to the ability to detoxify rishitin [82]. This knowledge could be used to develop inhibitors of phytoalexin-detoxifying enzymes as part of a phytosanitary treatment based on supporting self-defense of the plant. Alternatively, resistant plants that produce several different phytoalexins could be generated by either classical breeding or biotechnological methods. However, this approach requires additional knowledge on the genes and regulators involved in phytoalexin biosynthesis.

Identification of genes involved in phytoalexin generation is facilitated by the elucidation of the maize and sorghum genome sequences [37,83]. The sequence information now allows prediction of target genes that then need to be functionally characterized. One of the challenges lies in the identification of genes specific for phytoalexin production that do not lead to the generation of unplanned derivatives with a potentially adverse effect on human health.

To characterize gene function and study the impact of phytoalexins in plant resistance, the generation of plants that either do not express or overexpress phytoalexins will be a great help. Generation of recombinant plants is still a major challenge in research on maize and sorghum gene function. However, several techniques have been successfully employed and may accelerate the gain of knowledge. In addition to the laborious search for mutants in plant libraries generated by chemical mutagenesis using TILLING [84,85], Virus induced gene silencing (VIGS) was successfully applied to transiently silence genes in maize [49] and sorghum [86]. Sorghum transformation via microparticle bombardment of undifferentiated cells has been done [87], as well as new tools developed for the generation of targeted gene knockouts. Among these promising tools are the use of zinc finger nucleases (ZFNs) [88], TAL effector nucleases (TALENs) [88,89] and, most recently, the use of the clustered regulatory interspersed short palindromic repeat (CRISPR)/CRISPR-associated protein (Cas) system [90,91]. The newly generated plants would need to be subsequently tested for pathogen resistance under real-life conditions to learn how environmental stress affects plant protection by phytoalexins. In addition to new tools for generation of targeted gene disruption lines, mRNA sequencing of infected plants has been successfully used in maize and sorghum to discover target genes contributing to plant defense [30,31,92,93].

The 3-deoxyanthocyanidins have been proposed as medical agents against proliferation of several human cancer cell lines [94] and have been shown to induce apoptosis, inhibit cell proliferation, metastasis, and angiogenesis and sensitize tumor cells to therapeutic-induced cytotoxicity [95]. The flavone luteolin is well characterized for its antioxidant and anti-inflammatory activities both *in vitro* and *in vivo* [96]. Luteolinidin in a concentration of 200 µM reduced the viability of HL-60 cells by 90% and HepG2 by 50% [94]. Experiments using 3-deoxyanthocyanins against colon cancer stem cells showed a reduction of proliferation and apoptosis in these cells, in which luteolinidin was more effective than apigeninidin [97]. The compounds also presented effect against breast cancer MCF 7 cells [98] and were effective preventing the oxidation of LDL [99]. Moreover, sorghum extracts rich in 3-deoxyanthocyanidins were demonstrated to induce phase II enzymes [100], which are considered indicators of protection against carcinogens in animal cells [101]. An increased knowledge of the mode of action of phytoalexins and related compounds will help to explore their potential for use in human health.

In addition to their potential use in cancer treatment, other beneficial uses for the sorghum-specific phytoalexins have been proposed. These include the use as natural and persistant hair and food colorants [102,103], since they are natural products that have high color stability at various pH values, temperatures and light intensities [104]. In order to facilitate industrial production, a new sorghum variety *REDforGREEN* (*RG*) was recently developed using mutagenesis-assisted breeding. This variety overexpresses 3-deoxyanthocyanidins in leaf tissues, which highly increases the yield of pigments [103].

## 11. Striving for the Trophy: Challenges Ahead

Great efforts have resulted in a wealth of information on the identity, inducing conditions, and the biosynthesis genes of the major phytoalexins in both sorghum and maize. However, for some of the compounds, biosynthesis pathways are not completely elucidated. Although genes can be predicted, their involvement in phytoalexin biosynthesis is unclear. Once their involvement is assured, the enzymes can be tested for their catalytic abilities to know how they contribute to the pool of different phytoalexins. In addition to the identification of the remaining biosynthesis genes, their regulation will need to be studied. Different regulators and inducing molecules are expected to function in orchestrating sequential phytoalexin accumulation. Finally, we need to learn more about the mechanism of toxicity towards fungi, bacteria or insects, in order to be able to use phytoalexins for the creation of resistant crop plants. Future research on these promising compounds will help to preserve world nutrition and improve world economy in many different aspects.
